# Plasmalogens and Alzheimer’s disease: a review

**DOI:** 10.1186/s12944-019-1044-1

**Published:** 2019-04-16

**Authors:** Xiao Q. Su, Junming Wang, Andrew J. Sinclair

**Affiliations:** 1 Institute for Health and Sport, Victoria University, P.O. Box 14428, Melbourne, VIC 8001 Australia; 2 Department of Pathology, University of Mississippi Medical Centre, Jackson, MS 39213 USA; 3 Faculty of Health, Deakin University, Geelong, VIC 3217 Australia; 4 Department of Nutrition, Dietetics and Food, Monash University, Notting Hill, VIC 3800 Australia

**Keywords:** Plasmalogens, Alzheimer’s disease, Biomarker, Therapeutic efficacy, Mechanisms of action

## Abstract

Growing evidence suggests that ethanolamine plasmalogens (PlsEtns), a subtype of phospholipids, have a close association with Alzheimer’s disease (AD). Decreased levels of PlsEtns have been commonly found in AD patients, and were correlated with cognition deficit and severity of disease. Limited studies showed positive therapeutic outcomes with plasmalogens interventions in AD subjects and in rodents. The potential mechanisms underlying the beneficial effects of PlsEtns on AD may be related to the reduction of γ–secretase activity, an enzyme that catalyzes the synthesis of β-amyloid (Aβ), a hallmark of AD. Emerging in vitro evidence also showed that PlsEtns prevented neuronal cell death by enhancing phosphorylation of AKT and ERK signaling through the activation of orphan G-protein coupled receptor (GPCR) proteins. In addition, PlsEtns have been found to suppress the death of primary mouse hippocampal neuronal cells through the inhibition of caspase-9 and caspase-3 cleavages. Further in-depth investigations are required to determine the signature molecular species of PlsEtns associated with AD, hence their potential role as biomarkers. Clinical intervention with plasmalogens is still in its infancy but may have the potential to be explored for a novel therapeutic approach to correct AD pathology and neural function.

## Introduction

Plasmalogens are a subclass of phospholipids, mainly found in the cell membranes. Ethanolamine plasmalogens (PlsEtns) are the predominant phospholipids in the brain, kidney, lungs and skeletal muscle [1]. They are characterised by having a vinyl ether bond linking the fatty aldehyde to the glycerol molecule in the 1-position and a fatty acyl bond in the 2-position. There has been an increasing interest in plasmalogens in the last two decades due to their biological roles in the body and association with various diseases. It has been reported that reduced levels of PlsEtns in the plasma are associated with Alzheimer’s disease (AD), cardiovascular disease, hypertension, cancer and respiratory disease [1–7]. Several mechanisms associated with the action of PlsEtns have been suggested by various authors, through predominantly in vitro studies. These include helping maintaining membrane physical bilayer properties to facilitating membrane fusion and signal transduction processes, including cholesterol efflux [8, 9]; preventing oxidative stress; and reducing inflammatory response [7, 10–13].

This review focuses on the association(s) between plasmalogens and Alzheimer’s disease. AD is a complex neurodegenerative disease characterized by progressive memory loss and cognitive impairment associated with progressive neural cell loss predominantly observed in the hippocampus [14, 15]. The causes and mechanisms of the development of AD are not fully elucidated, although progressive accumulation of β-amyloid fibrils (Aβ plaques) and abnormal forms of tau (tau tangles) within and outside of neurons are considered to be the neuropathological hallmark [14, 15]. Currently, there is no established biomarker for AD to allow early detection of the risks for the disease and potential intervention to prevent disease progression [16]. Although available anti-dementia agents have shown some impacts on AD in a limited period of time, more desirable therapeutic agents with potent efficacy and long-lasting effects are needed.

The aim of this review is to provide an overview of current knowledge of the biology of PlsEtns with an emphasis on their association with AD. We briefly discuss the chemical and biological properties of PlsEtns; and present the most recent literature evidence on their association with AD, their mechanisms of action and potential use as a diagnostic and prognostic biomarker and new therapeutic targets for AD.

## Properties of Plasmalogens

**Fig. 1:**
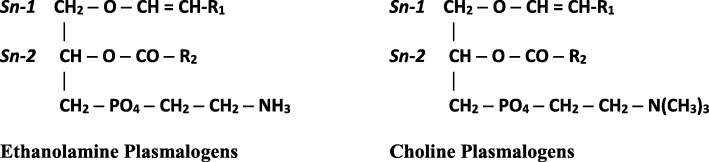
Chemical structures of Plasmalogens. R_1_ = palmitic acid (16:0) or stearic acid (18:0) or oleic acid (18:1). R_2_ = arachidonic acid (20:4) or docosahexaenoic acid (22:6) or oleic acid (18:1) or linoleic acid (18:2). When there is no double bond in the alkyl chain in the 1-position, the plasmalogens are referred to as alkyl plasmalogens, in contrast to when there is a double bond, as shown here, and they are referred to as alkenyl plasmalogens

Plasmalogens are subclass of phospholipids characterized by the presence of a vinyl ether bond at the sn-1 position and an ester bond at the sn-2 position of a glycerol backbone. The sn-1 position consists of C16:0 (palmitic acid), C18:0 (stearic acid) or C18:1 (oleic acid) carbon chains, and the head group is usually either ethanolamine or choline, thus there are two predominant types of plasmalogens, ethanolamine plasmalogens (PlsEtns) and choline plasmalogens (PlsChos). The sn-2 position is predominately occupied by a polyunsaturated fatty acid, specifically arachidonic acid (ARA) or docosahexaenoic acid (DHA) [1–3, 17] (Fig. 1). Plasmalogens are found in almost all mammalian tissues, although the highest concentrations are found in brain, red blood cells, skeletal muscle and spermatozoa and can represent as much as 18–20% of the total phospholipids in cell membranes [1, 15]. Their distribution and content varies significantly in different tissue/cell types, with PlsEtns 10-fold higher than PlsChos except in muscle [18]. Brain has the highest content of PlsEtns and it constitutes 30 mol% of total phospholipids in human brain, whereas heart muscle has a higher content of PlsChos [3, 7, 17]. Moderate amounts of plasmalogens are found in kidney, skeletal muscle, spleen and blood cells, while liver shows low plasmalogen content [2]. The unique functions of plasmalogen species in the body are directly related to the property of the sn-1 vinyl ether bond and the enrichment of polyunsaturated fatty acids at the sn-2 position [1].

**Fig. 2:**
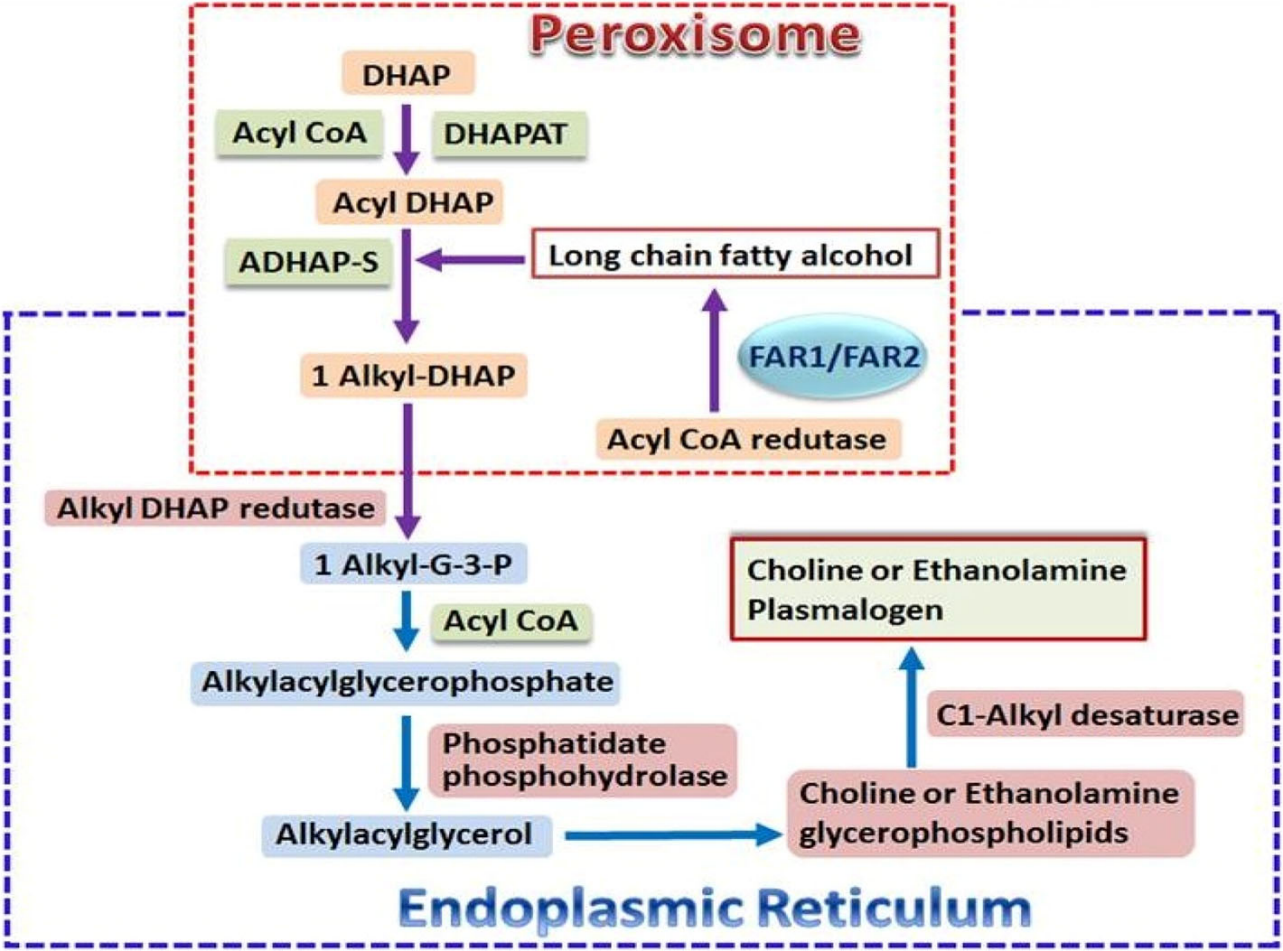
Biosynthetic pathway of Plasmalogens. Abbreviations: DHAP, dihydroxyacetone phosphate; DHAPAT, dihydroxyacetone phosphate acyltransferase; DHAP, dihydroxyacetone phosphate; ADHAP-S, alkyl dihydroxyacetone phosphate synthase; FAR1/2, acyl-CoA reductase 1 and 2

Phospholipids are either provided directly from the diet or via de novo synthesis. Dietary phospholipids are typically consumed in low amounts, about 2–5 g per day, through the intake of almost all foods, especially eggs, soybean, meat, fish, milk and other dairy products [19, 20]. Dietary plasmalogens are absorbed in the intestine and delivered to tissues [21] although the details of digestion, absorption and subsequent transport of dietary plasmalogens are essentially unknown. A similar situation prevails for absorption and subsequent transport of dietary diacyl phospholipids where specific details are still far from clear [22, 23]. Circulating plasmalogens are either derived from dietary sources and/or are synthesized mainly in the liver and gastrointestinal epithelium [24, 25] and are exported into the blood via transport proteins of which low density lipoprotein (LDL) is a major carrier [26]. From there they are transported to the brain via a LDL receptor-mediated transcytosis pathway [27]. Plasmalogens are not only important structural phospholipids in the cell membranes, they are also reservoirs of secondary messages and mediators of membrane dynamics and involved in membrane fusion, ion transport, cholesterol efflux, membrane-bound enzyme activity, and diffusion of signal-transduction molecules. The biological functions of plasmalogens have been well documented in the literature [1, 2, 7–9, 28–30]. Plasmalogens are also antioxidants, and have been shown to have a protective role against oxidative stress, especially in the brain and heart [12]. The biosynthesis of plasmalogens is initiated in peroxisomes and terminated in the endoplasmic reticulum (Fig. 2) [7, 31]. Defects occurring at the synthetic process may affect their biological functions in the body. Inherited (primary) plasmalogen deficiency is rare (1/100,000) and has been found to be associated with peroxisomal disorders resulting from mutations /defects in the genes involved in the synthesis of peroxisomal protein transporter or enzymes required for plasmalogen syntheses [1]. Primary plasmalogen deficiency associated with the inherited human peroxisomal disorder, rhizomelic chondrodysplasia punctata (RCDP) shows deficiency of tissue plasmalogens and results in severe disorders in multiple organs such as brain, bone, lens, kidney and heart [17]. Secondary deficiency of plasmalogens resulting from decreased synthesis and/or increased degradation of plasmalogens, was reported to be associated with metabolic and inflammatory disorders such as cardiac diseases, diabetes mellitus, cancer, respiratory disease and Alzheimer’s disease [1–3, 7, 17]. Choline plasmalogens play an important role in cardiac tissue, but represent a minor species in most other organs thus most likely an insignificant role in those organs.

## Alzheimer’s disease

AD is a complex neurodegenerative disease characterized by progressive memory loss and cognitive impairment [14]. It is the most common cause of dementia in older people. It is associated with progressive neural cell loss predominantly observed in the hippocampus. The cause and mechanism of AD has not been fully elucidated, although progressive accumulation of β-amyloid fibrils (Aβ plaques) and abnormal forms of tau (tau tangles) within and outside of neurons are considered to be the neuropathological hallmarks [14, 15, 32–34]. A number of other neuropathologies of AD have also been suggested including neuronal shrinkage, hypomyelination, neuroinflammation, mitochondrial oxidative stress, endoplasmic reticulum stress, and cholinergic dysfunction [1, 3, 35–37]. Various risk factors promote pathological changes well before the onset of clinical symptoms of AD and this includes age; cardiovascular risk; lifestyle related factors such as obesity, diabetes, depression, smoking, and insufficient diet; family history and possibly genetic factors; environmental factors (aluminium and or zinc accumulation in the brain), serious head injury [1, 14]. It is estimated that the prevalence of AD may reach to 74 million worldwide by 2030 [38], therefore early diagnosis and effective treatment are crucial in order to reduce the incidence of the disease. However, as the pathology of AD begins well before symptoms are manifested, the progressive neurodegeneration and neural cell loss in the brain are often neglected until impairments in cognitive performance become noticeable [14]. When the loss of neuronal cells reaches a critical point with cognition deficit, approximately 50 to 80% of cells have already died, a condition that the course of the disease progression becomes difficult or impossible to be changed. Currently, there are no established biomarkers for AD to allow early detection of the risks for the disease and potential intervention to prevent disease progression [16]. Among several factors being studied as possible biomarkers for AD are the amyloid-β in the brain as shown in positron emission tomography (PET) imaging and levels of certain proteins in fluid (for example, levels of Aβ and phosphorylated tau, a major microtubule associated protein of a mature neuron) in the cerebrospinal fluid and levels of particular groups of proteins in blood) [14]. However, those tests are either invasive or costly. The sensitivity of those tests also reflect imperfection for clinical diagnosis [39]. There is also no effective treatment for AD although some anti-dementia agents such as cholinesterase inhibitors and Memantine (Namenda) have shown some improvements in cognition, global function and /or activities of daily living in some patients with AD for a limited period of time [40, 41].

## Association of PlsEtns with Alzheimer’s disease and their potential use as a biomarker

There are increasing numbers of studies in the last two decades which have reported a direct link of plasmalogen deficiency and AD. Since the first study showing a significant deficiency of PlsEtns relative to PE in the post-mortem brain samples of AD patients was published in 1995 [42], much attention has been devoted to PlsEtns and their association with AD. Recent studies have predominately focused on the potential therapeutic role of PlsEtns and the molecular mechanisms underlying their beneficial effects. Table 1 summarises key studies focusing on the relation of PlsEtns and AD, in particular those published in the last 5 years.

**Table 1:** Plasmalogen (Pls) deficiency and their therapeutic use in AD and related disorders, and emerging molecular evidence on their neuronal protection role

Author	Design	Objectives	Outcomes
	Human studies		
Fujino et al. 2017	Patients (aged 60–85 years) with mild AD and mild cognitive impairment (MCI) were randomized to a multi-center, double-blind, placebo-controlled trial to receive 24 weeks of treatment with either: 1 mg/d purified plasmalogens (Pls) extracted from scallops (*n* = 140), or placebo (*n* = 136)	To test the efficacy of oral administration of Pls on cognitive function and blood Pls changes in patients with mild AD and MCI. Primary outcome: Mini Mental State Examination-Japanese (MMSE-J); secondary outcomes: Wechsler Memory Scale-Revised (WMS-R), Geriatric Depression Scale-Short Version-Japanese (GDS-S-J), and concentration of PlsEtns in circulation.	Oral administration of Pls significantly improved memory among female mild AD patients and those aged below 77 years as shown in WMS-R test. Mild AD patients showed a significantly greater decrease in plasma PlsEtns in the placebo group than in the treatment group.
Wood et al. 2016	Serum samples of clinical diagnosed Late-Onset AD (LOAD) patients (*n* = 90), patients with mild cognitive impairment (MCI, *n* = 77), and controls (*n* = 51) aged 76 = 78 were analyzed by lipidomics.	To investigate the levels of PlsEtns and diacylglycerols utilizing high resolution mass spectrometry, and correlate the lipid levels with disease.	Three patient cohorts within each clinical diagnosis (LOAD and MCI were observed: lower circulating PlsEtns; higher circulating diacylglycerols; and neither of these two lipid alterations. More patients showed low level of PlsEtns in advanced stage of disease.
Yamashita et al. 2016	Plasma and red blood samples of 28 AD patients (age: 72.5 ± 1.4) and 28 normal control subjects (age: 74.1 ± 1.3) were analyzed. Evaluation of plasma Aβ was correlated with phosphatidylcholine hydroperoxide (PCOOH), and PlsEtn in the blood of patients with AD.	To investigate the interaction of Aβ, peroxidation of phosphatidylcholine and PlsEtn in AD patients.	Plasma from patients with AD showed lower concentrations of PlsEtn species, especially DHA-containing PlsEtn. In addition, lower PlsEtn and higher PCOOH levels were observed in red blood cells (RBC) of AD patients. In both AD and control blood samples, PCOOH levels of RBC tended to correlate with plasma levels of Aβ40.
Wood et al. 2015	Lipidomics analysis of post-mortem cerebrospinal fluid (CSF), frontal cortex grey matter, and subjacent white matter	To define potential biomarkers that distinguishes cognitively intact subjects from those with incipient or established dementia; and understands the role of brain lipids in pathophysiology of aging and age-related cognitive impairment.	Monoacylglycerols (MAG), diacylglycerols (DAG), and fatty acid 26:0 were elevated in the grey matter of the mild cognitive impairment (MCI) and old dementia (OD) cohorts. PlsEtns were decreased in the grey matter of the young dementia (YD) and OD cohorts while and phosphatidylethanolamines were lower in the MCI, YD and OD cohorts.
Wood et al. 2010	The serum PlsEtns of 40 AD patients and 66 controls aged 67–89 years were analyzed and correlated with cognitive functions using AD assessment scale-cognitive (ADAS-Cog). Serum PlsEtns of AD patients were retested 1 year later.	To evaluate the relation between the level of circulating PlsEtns and cognitive function using ADAS-Cog in AD patients in comparison with controls.	Only subjects with serum DHA-PlsEtn ≤75% or less of normal levels exhibited cognitive decline over a 12 month period. There was no change in ADAS-Cog scores among participants with normal serum PlsEtn levels at baseline (> 75%).
Goodenowe et al. 2007	The serum samples of 324 dementia subjects (3 groups: low, moderate and severe cognitive impairments) were analyzed and compared with 68 cognitive normal subjects aged 50–90 years to investigate the relations between dementia severity and PlsEtns levels. 209 healthy subjects aged 50–95 years were divided into 3 age groups (50–59, 60–69, and 70–95 years) and their serum were analyzed to determine the effect of age on PlsEtns levels. Postmortem serum samples of subjects with AD (*n* = 20) and those without AD pathology (*n* = 19) were analyzed to compare the serum PlsEtns levels. Serum from 50 clinically diagnosed AD subjects (3 stages), who were later confirmed to have AD upon postmortem examination were analyzed to determine the DHA PlsEtns levels. Serum samples of 80 Japanese AD subjects and 80 non-demented Japanese subjects living in Japan were analyzed to determine the effect of ethnic or environmental differences on serum DHA PlsEtns levels in AD	To determine PlsEtns depletion in the brain of subjects with AD; and whether decreased brain levels of PlsEtns in AD are a centrally mediated effect caused by Aβ accumulation or there are much broader changes occurred.	The levels of PlsEtns species were significantly reduced in all three groups of AD subjects, and the decrease was correlated with the severity of AD. Peroxisome-derived PlsEtns was also significantly reduced in all stages of AD. Subject % with very low PlsEtns in the 60–69 year cohort was more than double than other two groups. ↑ mortality rate in both AD subjects and peroxisomal disorders with ↓ PlsEtns. Serum PlsEtns levels were significantly reduced in the postmortem (55%) AD subjects, and clinically diagnosed AD subject (47%). The decrease was related to the severity of disease. Serum DHA-PlsEtns levels were significantly reduced in the AD subjects.
Han et al. 2001	Post-mortem human brain tissue and mouse brain tissues from two animal models of AD, APPV717F and APPsw were analyzed using Electrospray ionization mass spectrometry (ESI/MS)	To examine systematically plasminogen content in cellular membranes of gray and white matter from different regions of human subjects with a spectrum of AD clinical dementia ratings	A dramatic decrease in PlsEtns content (up to 40 mol% of total PlsEtns) in white matter at a very early stage of AD; a correlation of the deficiency in gray matter PlsEtns content (10 mol% - 30 mol% deficiency) with the severity of AD (very mild to severe); no alterations of PlsEtns content and molecular species in cerebellar gray matter despite dramatic alterations of PlsEtns content in cerebellar white matter. 10 mol% deficiencies was present in mice at age of 18 months in cerebral cortices but not in cerebella.
	Animal studies		
Katafuchi et al. 2012	Male C57/6 J mice (10 months old) were randomly divided into 3 groups: Pls (20 mg/kg) + lipopolysaccharide (LPS), LPS, and control. Pls were extracted from chicken breast muscle. All treatments were for 7 days.	To elucidate the effects of Pls on neuroinflammation and β-amyloid proteins accumulation in the hippocampus, and changes in hippocampal Pls content following peripheral administration of LPS in adult mice.	Pls administration suppressed the activation of glial cells (microglia) induced by LPS, indicating attenuation of neuroinflammation in the hippocampus. Pls treatment also abolished β-amyloid proteins in the hippocampus; and suppressed the reduction of Pls contents in hippocampus induced by LPS.
Mawatari et al. 2012	20 male Zucker diabetic fatty (ZDF) rats aged 4 weeks were randomly divided into 2 groups (10 per group) and fed with either 0.1% PlsEtn or control diet for 4 weeks. In addition, 18 male Wistar rats aged 6 weeks were divided evenly into 2 groups and fed with PlsEtn or control diet for 9 weeks.	To examine the health effects of dietary PlsEtn.	Supplementation with 0.1% PlsEtn in both Zucker diabetic fatty rats and Wistar rats reduced the plasma cholesterol and phospholipids. Correspondingly, erythrocyte PlsEtn and phosphatidylethanolamine were increased.
	In vitro studies		
Hossain et al. 2016	Mouse neuroblastoma derived cells, (Neuro 2A, or N2A), astrocyte cell lines (A1) and microglial cell lines (MG6) were treated with Pls extracted from chicken skin (96.5% PlsEtns). Primary hippocampal neurons were prepared from E-18 embryo of mice.	To investigate how PlsEtns enhance AKT and ERK signaling and prevent neuronal cells.	PlsEtns activated orphan GPCR (G-protein coupled receptor) proteins to induce ERK signaling in neuronal cells. Overexpression of GPCRs enhanced PlsEtns-mediated phosphorylation of ERK and Akt in cells. The GPCRs-mediated cellular signaling was reduced significantly when the endogenous PlsEtns were reduced.
Hossain et al. 2013	Neuroblastoma derived cells Neuro-2A and astrocyte-derived cells A1 were treated with Pls (96.5% PlsEtns).	To investigate the molecular mechanism behind the neuronal protection of PlsEtns against apoptotic stimuli.	PlsEtns prevent neuronal cell death by enhancing phosphorylation of AKT and ERK signaling in neuronal cells. They also inhibited primary mouse hippocampal neuronal cell death induced by nutrient deprivation which was associated with the inhibition of caspase-9 and caspase-3 cleavages.
Onodera et al. 2014	*γ* -secretase activity was measured in an in vitro assay using yeast microsomes and reconstituted liposomes.	To investigate the effect of PlsEtns on *γ*--secretase activity in vitro.	PlsEtns reduced *γ*--secretase activity. Bacterial PlsEtns preparation showed dose-dependent inhibition of *γ* -secretase activity.
Rothhaar et al. 2012	SH-SY5Y cells, and 58 postmortem brain samples from 37 AD patients and 21 controls aged 61–88 years were analyzed for *γ*-secretase activity. For ex vivo analysis of *γ*-secretase activity postnuclear fractions, 6 additional human postmortem brains and brains of C57BI6/N wildtype mice were also analyzed.	To determine whether PlsEtns are able to modulate amyloid precursor protein (APP) processing or if the reduced PlsEtns level is a consequence of AD.	PlsEtns levels were reduced in postmortem AD brains. PlsEtns directly reduced *γ*-secretase activity in SH-SY5Y cells, postmortem AD brains and mouse brains. Protein and RNA level of the secretases were unaffected.

It has been demonstrated that decreased levels of PlsEtns have not only found in the post-mortem brain samples [43–46], but also in cerebrospinal fluid [43, 47], plasma, serum and red blood cells of AD patients [30, 48–50]. A 70% reduction of PlsEtns has been observed in the brain of AD patients compared with healthy brain tissues [30, 32]. The deficiency of PlsEtns in neurodegeneration was found to be specific to AD and not observed at the primary site of neurodegeneration in Huntington’s disease nor Parkinson’s diseases [3, 44]. It was also reported that more remarkable decrease of PlsEtns was observed in the neurodegeneration sites such as hippocampus, temporal cortex and frontal cortex, but not the cerebellum of AD brain [15, 42, 44]. Gray matter PlsEtns was found to have different fatty acid composition from white matter PlsEtns at sn-2 position. In white matter, the sn-2 position is dominated by oleic acid while in gray matter docosahexaenoic acid (DHA, 22:6) and arachidonic acid (20:4) predominate [3]. Furthermore, reduction of PlsEtns in different brain tissues has been reported to be associated with different stage of AD progression. White matter PlsEtns deficiency was found to be associated with early stage of disease, and a dramatic decrease of up to 40 mol% of the total PlsEtns has been observed in the post-mortem AD brain [44, 51]. This decrease of PlsEtns was not correlated with cognition functions [3]. While gray matter PlsEtns deficiency has shown a correlation with the severity of disease, with ~ 10 mol% - 30 mol% of the total PlsEtns reduction being recorded in the post-mortem AD brain corresponding to very mild and severe disease status [3, 44]. Consistently, a ≤ 75% decrease of serum plasmalogens levels in AD patients compared with age-matched controls has also shown an association with a cognitive function decline [30]. There have been many reports showing that DHA is related closely to brain functions [52–56]. Therefore, the association of gray matter PlsEtns and white matter PlsEtns with different stage of AD may be attributed at least in part to the discrepancy in their fatty acid compositions at sn-2 position, and/or their major functions in neuronal cells compared with myelin. Correspondingly, observations have shown that levels of DHA and DHA-containing PlsEtns were significantly reduced in the brain, liver, plasma and serum of AD patients and the extent of decrease was correlated with cognitive deficit in AD patients [3, 24, 30, 49]. Furthermore, the severity of disease was improved when circulating levels of DHA and plasmalogens were high, particularly PlsEtns containing DHA at sn-2 [30, 49].

The cause of PlsEtns deficiency in AD brain is not clear. It is also not known whether the decrease of PlsEtns in the patients with AD is the cause or the consequence of the disease. It may be both [15], although an earlier suggestion indicated that it may be the cause of the ethology of AD [57]. A few possible mechanisms with regards to the decrease of PlsEtns in AD have been suggested including peroxisome dysfunction, oxidative stress, alterations in membrane lipid rafts and inflammatory responses [2, 3, 42, 58, 59]. Plasmalogens synthesis is initiated in peroxisomes [31], and therefore changes/damages in peroxisome would result in alterations in plasmalogen synthesis. Peroxisome deficits have been reported in the liver and brain of AD subjects [24, 58, 60]. A correlation was also observed between increased very long chain fatty acids (VLCFAs: behenic acid C22:0, lignoceric acid C24:0 and hexacosanoic acid C26:0), decreased plasmalogens in AD brain, and increased peroxisome volume density in neuronal cells [58]. All of these VLCFAs are metabolized in peroxisomes, thus these data again support peroxisome dysfunction in AD [3]. Furthermore, changes in VLCFAs and PUFA- containing plasmalogens also show impacts on functional performance. An increase of VLCFAs in the cortex, and decrease in DHA-containing plasmalogens in brain, liver and plasma was found to be associated with cognitive deficit in AD patients [24, 30, 44, 58].

Loss of PlsEtns in the AD brain could also be related to oxidative stress, leading to plasmalogen degradation by reactive oxygen species (ROS) [1]. The presence of a vinyl–ether bond makes plasmalogens more susceptible to oxidative stress [61]. This suggests that plasmalogens may act as scavengers to protect other lipids and lipoproteins from oxidative damages [11, 62]. The antioxidant effect of plasmalogens has been reported towards a wide range of ROS [10, 59]. Reduced plasmalogens might further enhance ongoing oxidative damage in AD, and alter membrane properties to promote further damage [1]. The lipid environment in the cell membrane could affect amyloid precursor protein processing through processing enzymes as they are integral membrane proteins and the Aβ cleavage takes place within the membrane. In addition, increased membrane free cholesterol associated with PlsEtns deficiency increased the production of Aβ from amyloid precursor proteins [63]. Thus decreased PlsEtns in AD may facilitate Aβ production. Furthermore, one in vitro study has shown that Aβ aggregation can be modulated by plasmalogens [64]. On the other hand, it has also been shown that increased Aβ and ROS reduced the expression of a rate-limiting enzyme, alkyl-dihydroxyacetone phosphate-synthase, for plasmalogens de novo synthesis, due to the dysfunction of peroxisomes where plasmalogens are biosynthesized, resulting in a decrease in plasmalogen level [60]. Finally, since PlsEtns are major endogenous lipid constituents that facilitate membrane fusion of synaptic vesicles associated with neurotransmitter release, loss of PlsEtns might be expected to adversely affect synaptic structure and function, thus potentially contributing to the synaptic dysfunction and neurotransmitter depletion observed in AD [1, 3, 59].

**Fig. 3:**
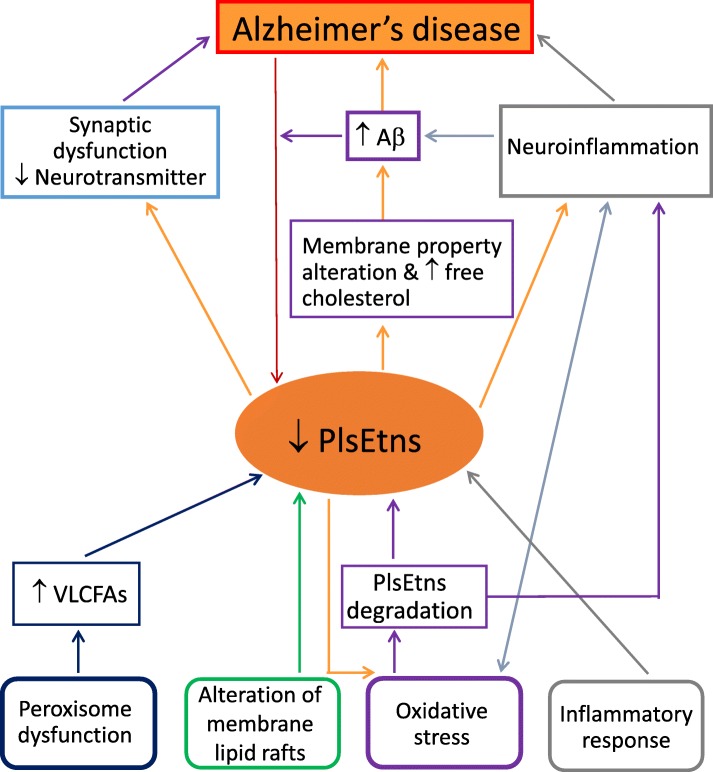
Proposed mechanistic association of ethanolamine plasmalogens deficiency and Alzheimer’s disease. Abbreviations: Aβ, β-amyloid; PlsEtns, ethanolamine plasmalogens; VLCFAs, very long chain fatty acids. PlsEtns have a close association with Alzheimer’s disease (AD). Decreased levels of PlsEtns have been commonly found in AD patients, and are correlated with cognition deficit and severity of disease, although it is not known whether it is the cause or the consequence of the disease. It has been suggested maybe it is both. A few possible mechanisms with regards to the decrease of PlsEtns in AD have been suggested: peroxisome dysfunction, oxidative stress, alterations in membrane lipid rafts and inflammatory responses. Decreased PlsEtns may further enhance oxidative damage and alter membrane properties in AD. This plus increased membrane free cholesterol associated with PlsEtns deficiency could increase the production of Aβ. Aβ and reactive oxygen species could further decrease PlsEtns level. PlsEtns are major lipids facilitating membrane fusion of synaptic vesicles associated with neurotransmitter release, thus loss of PlsEtns could potentially contribute to the synaptic dysfunction and neurotransmitter depletion in Alzheimer’s disease. The association of decreased level of PlsEtns and neuroinflammation may be related to antioxidant properties of plasmalogens that protect cells from oxidative stress. Neuroinflammation has been reported to be associated with Aβ accumulation

Decreased levels of plasmalogen have been observed in neuroinflammation and that might lead to a diminished level due to the antioxidant properties of plasmalogens that protect cells from oxidative stress [59]. Considerable evidence has suggested that there is a connection loop between neuroinflammation, Aβ accumulation, ROS production, and plasmalogen deficiency [59]. Furthermore, degradation of PlsEtns by the enzyme, plasmalogen-selective phospholipase A2 (Pls-PLA2), releases DHA or arachidonic acid from the *sn*-2 position of the glycerol backbone, and this process is possibly activated by ceramide produced under inflammatory conditions which might contribute to the loss of PlsEtns in the brain [65, 66]. Arachidonic acid is a substrate for the synthesis of prostaglandins, thromboxanes and leukotrienes. Metabolites derived from DHA include resolvins, maresins, docosatrienes and neuroprotectins [1, 67]. All of these molecules regulate inflammatory responses, with arachidonic acid and its derivatives involved in pro-inflammatory processes and DHA and its derivatives involving in anti-inflammatory processes [13, 55, 68] (Fig. 3).

An increasing number of studies have shown that PlsEtns deficiency is associated with AD pathology. These findings have led the researchers to propose a role of these molecules as a potential biomarker for AD. However, since decreased levels of circulating plasmalogens are also found in a number of other clinical disorders, including ischemic cerebrovascular disease, hypertension, uremia, and hyperlipidemia, the utility of PlsEtns as a biomarker for AD needs to be further studied, as suggested by Wood [3, 50]. Taken together, future studies should focus on the determination of signature molecular species of PlsEtns associated with AD. This may provide specific evidence on the efficacy of PlsEtns as a reliable biomarker for AD.

## Plasmalogens as a potential therapy for AD

Several researches have focused on the plasmalogens replacement therapy in animals, and reported promising outcomes. Katafuchi et al. [59] reported that intraperitoneal administration of purified plasmalogens for 7 days attenuated the neuroinflammation in the hippocampus of adult male C57/6 J mice, which had been induced by lipopolysaccharide. Plasmalogen treatment also abolished Aβ protein accumulation in the hippocampus, and this was correlated with the suppressed reduction of the plasmalogen content in the hippocampus [59]. Feeding a test diet containing 10 wt% of phospholipids to rats for 7 days has been found to increase plasmalogen concentration by threefold in blood plasma and by 25% in the liver [21]. Supplementation with 0.1% PlsEtn for 4 weeks in Zucker diabetic fatty (ZDF) rats and for 9 weeks in healthy Wistar rats reduced the plasma cholesterol and phospholipid concentrations; corresponding with this, an increase in erythrocyte PlsEtn and phosphatidylethanolamine was observed [17]. Oral administration of plasmalogens precursor PPI-1011 (100 mg/kg/day) to C57/6 J mice for 2 weeks restored the reduced levels of PlsEtns in plasma and brain, and this plasmalogen restoration was associated with a stimulated re-myelination of neuronal cells [57].

Little information is available on the therapeutic efficacy of plasmalogens in humans, suggesting a need for future research. A recent randomized, double-blind, placebo-controlled clinical study [15] reported a significant improvement in memory (as shown in Wechsler Memory Scale-Revised test) among female patients with mild AD aged below 77 years, after 24 weeks of oral administration with 1 mg/day of purified plasmalogens extracted from scallops. The study was conducted in multiple centres with a total of 276 patients (140 in the treatment group and 136 in the placebo group) aged 60 to 85 years. Correspondingly, the plasma PlsEtn in mild AD patients showed a significantly greater decrease in the placebo group compared with the treatment group.

The molecular mechanisms underlying the beneficial effects of PlsEtns on AD have been explored predominantly through in vitro studies. It was found that plasmalogens strongly reduce activity of γ–secretase, a membrane-associated aspartic protease that catalyzes the final step of Aβ synthesis, i.e. produce several Aβ peptides of different lengths such as Aβ40, Aβ42 and Aβ43 [32, 46]. Increased aggregation of Aβ42 and Aβ43 can result in the deposition of Aβ and is a common outcome of the familial AD [69]. Studies have also shown that PlsEtns prevented neuronal cell death by enhancing phosphorylation of protein kinase B (AKT) and extracellular signal-regulated kinase (ERK) signaling in neuronal cells, and this was through the activation of orphan GPCR (G-protein coupled receptor) proteins. Over-expression of GPCRs enhanced plasmalogen-mediated phosphorylation of ERK and AKT in neuronal cells, while GPCRs-mediated cellular signaling was reduced significantly when the endogenous PlsEtns levels were reduced [70, 71]. Furthermore, the inhibitory effect of PlsEtns on the death of primary mouse hippocampal neuronal cells was found to be associated with the inhibition of caspase-9 and caspase-3 cleavages, indicating the anti-apoptotic action of PlsEtns in the brain [71]. More in-depth animal studies are required to explore further the molecular mechanisms associated with the actions of PlsEtns in AD in order to gain a better understanding of these molecules and to evaluate their use as an effective therapeutic approach to correcting AD pathology and function.

## Conclusion

Many studies have shown that there is a link between PlsEtns deficiency and AD, although it is not clear whether the decrease of PlsEtns in the patients with AD is the cause or the consequence of the disease. Reductions of PlsEtns levels have been reported in plasma, serum, cerebrospinal fluid and brain tissue of AD patients. This suggests PlsEtns might be a candidate as a potential AD biomarker. However, due to the fact that decreased levels of circulating PlsEtns are also found in a number of other clinical disorders, further in-depth investigations are required to determine the signature molecular species of PlsEtns associated with AD. Little is known about the clinical efficacy of plasmalogens on AD treatment, and more intervention studies with PlsEtns replacement therapy in AD patients are warranted given the promising outcomes in animal studies. Future in vivo studies of molecular mechanisms associated with actions of PlsEtns would also help to determine the potential use of PlsEtns as a new therapeutic agent for AD.

## Abbreviations

AD: Alzheimer’s disease
ADHAP-S: Alkyl dihydroxyacetone phosphate synthase
AKT: Protein kinase B
Aβ: β-amyloid
DHA: Docosahexaenoic acid
DHAP: Dihydroxyacetone phosphate
DHAPAT: Dihydroxyacetone phosphate acyltransferase
ERK: Extracellular signal-regulated kinase
FAR1/2: Acyl-CoA reductase 1 and 2
GPCR: G-protein coupled receptor
LDL: Low density lipoprotein
PET: Positron emission tomography
PlsChos: Choline plasmalogens
PlsEtns: Ethanolamine plasmalogens
ROS: Reactive oxygen species
VLCFAs: Very long chain fatty acids
